# The multifaceted role of NF-κB signaling in psoriasis: from inflammatory amplification to epidermal remodeling

**DOI:** 10.3389/fimmu.2026.1808775

**Published:** 2026-07-01

**Authors:** Wenqin Cao, Xiaobo Hu, Yin Wang, Sheng Wei

**Affiliations:** Department of Dermatology, Jiashan First People’s Hospital, Jiashan, Zhejiang, China

**Keywords:** inflammatory networks, innate and adaptive immunity, keratinocytes, NF-κB signaling, psoriasis

## Abstract

Psoriasis is a chronic immune-mediated inflammatory skin disease characterized by persistent epidermal hyperplasia and relapsing inflammation. Although extensive progress has been made in elucidating individual pathogenic pathways, a unifying framework that integrates innate immune activation, adaptive immune maintenance, and keratinocyte-driven amplification remains incomplete. Accumulating evidence indicates that nuclear factor-κB (NF-κB) functions as a central convergence hub that coordinates these diverse inflammatory inputs in psoriasis. In this review, we synthesize recent advances demonstrating how innate immune sensors, including nucleic acid–sensing pathways and Toll-like receptors, converge on NF-κB to initiate inflammatory programs, and how adaptive immune circuits—particularly Th17/IL-17 signaling—exploit NF-κB to sustain disease chronicity. We further highlight the active role of keratinocytes as intrinsic amplifiers of inflammation through NF-κB–dependent programs governing hyperproliferation, oxidative stress responses, and secondary cytokine production. Beyond canonical signaling, emerging evidence reveals that non-coding RNAs and epigenetic regulators fine-tune NF-κB activity, shaping transcriptional persistence and inflammatory memory in psoriatic skin. Finally, we discuss therapeutic strategies targeting the NF-κB convergence hub, including upstream sensor antagonism, adaptor-level modulation, multi-target small molecules, natural products, and advanced skin-targeted delivery systems. By framing NF-κB as a networked regulatory hub rather than a linear pathway, this review provides an integrated perspective on psoriasis pathogenesis and highlights opportunities for precision immunomodulation with improved efficacy and safety.

## Highlights

- NF-κB acts as a convergence hub integrating innate immune triggers, Th17-driven adaptive responses, and keratinocyte-intrinsic inflammatory amplification in psoriasis.
- Keratinocytes actively shape disease persistence through NF-κB–dependent programs controlling hyperproliferation, oxidative stress, and secondary cytokine output.
- Targeting NF-κB networks via upstream sensors, subunit-specific regulation, and skin-localized delivery offers promising translational opportunities.

## 1 Introduction

Psoriasis is a chronic, immune-mediated inflammatory skin disorder characterized by erythematous, scaly plaques and a relapsing–remitting clinical course. Although the disease manifests primarily in the skin, accumulating clinical and experimental evidence indicates that psoriasis is not a purely cutaneous condition, but rather a systemic inflammatory disease with extensive immune involvement (1–6). Patients frequently exhibit extracutaneous comorbidities, including psoriatic arthritis, metabolic syndrome, cardiovascular disease, and psychological disorders, underscoring the systemic nature of the inflammatory burden. From an immunological perspective, psoriasis is marked by a paradoxical combination of localized tissue pathology and widespread immune dysregulation. Lesions are anatomically confined to the skin, yet they are sustained by continuous crosstalk among innate immune cells, adaptive immune compartments, and epidermal keratinocytes. This spatial confinement despite persistent immune activation suggests the existence of tightly coordinated, self-amplifying inflammatory circuits operating within the skin microenvironment.

Current pathogenic models converge on a three-center inflammatory framework (7–10). First, innate immune activation is initiated by dendritic cells and other innate sensors that respond to environmental stress, microbial signals, or endogenous danger-associated molecular patterns. These signals trigger the production of key cytokines and costimulatory molecules that prime adaptive immunity. Second, adaptive immune maintenance, dominated by Th17 cells and their signature cytokines such as IL-17A and IL-23, sustains chronic inflammation and reinforces immune memory within lesional skin (11–16). Third, keratinocytes function as active effectors and amplifiers, rather than passive targets, by responding to immune-derived cues with exaggerated inflammatory gene expression, hyperproliferation, and impaired differentiation. Through the release of cytokines, chemokines, and antimicrobial peptides, keratinocytes further recruit and activate immune cells, forming a self-perpetuating inflammatory loop (17–20). This integrated view highlights psoriasis as a systems-level inflammatory disease, in which innate immune triggers, adaptive immune reinforcement, and keratinocyte-intrinsic responses are dynamically interconnected rather than operating in isolation.

Within this complex inflammatory network, a central unanswered question is how diverse upstream signals originating from distinct immune compartments are integrated into a coherent and persistent transcriptional program. A growing body of evidence supports the notion that nuclear factor-κB (NF-κB) functions as a critical convergence hub that coordinates innate and adaptive immune signaling in psoriasis (21, 22). NF-κB signaling represents a common downstream endpoint for a wide range of pattern-recognition receptors, including Toll-like receptors and cytosolic nucleic acid sensors, as well as cytokine receptors engaged by IL-1 family members, TNF, and IL-17. In parallel, NF-κB activity is highly sensitive to cellular stress signals such as oxidative stress, metabolic perturbations, and hypoxia, all of which are prominent features of the psoriatic microenvironment (23, 24). Through these inputs, NF-κB integrates immune recognition, inflammatory signaling, and stress responses into a unified transcriptional output. Importantly, NF-κB signaling is not a monolithic pathway. Distinct upstream adaptors, signaling nodes, and NF-κB subunits can be selectively engaged in different cell types, leading to context-dependent inflammatory programs. In psoriasis, this flexibility enables NF-κB to coordinate innate immune activation in antigen-presenting cells, cytokine-driven amplification in Th17 cells, and inflammatory and proliferative responses in keratinocytes. As a result, NF-κB occupies a unique position at the interface of innate and adaptive immunity, translating heterogeneous extracellular cues into sustained tissue-specific inflammation. The therapeutic relevance of this convergence is increasingly apparent. Rather than acting as a simple pro-inflammatory switch, NF-κB operates as a network hub whose activity is shaped by upstream sensors, modulatory cofactors, epigenetic regulators, and noncoding RNAs. This network architecture creates multiple opportunities for selective intervention, allowing inflammatory amplification to be attenuated without globally suppressing immune function.

In this review, we adopt a convergence hub perspective to systematically examine how NF-κB integrates innate immune triggers and adaptive immune maintenance in psoriasis. By synthesizing recent mechanistic studies across immune cells and keratinocytes, we aim to clarify how NF-κB-centered signaling networks sustain chronic inflammation and to discuss emerging therapeutic strategies that target this hub with improved specificity and translational potential.

## 2 NF-κB signaling architecture and subunit-specific programs relevant to psoriasis

### 2.1 Canonical and non-canonical NF-κB signaling: key nodes and upstream receptors in psoriasis

The NF-κB family comprises a group of transcription factors that play central roles in immune regulation, inflammation, and tissue homeostasis. In mammalian cells, NF-κB signaling is classically divided into canonical (classical) and non-canonical (alternative) pathways, which differ in upstream triggers, signaling intermediates, and transcriptional outputs. Both pathways have been implicated in inflammatory skin diseases, although the canonical pathway predominates in psoriasis-associated inflammation. The canonical NF-κB pathway is rapidly activated by a broad spectrum of receptors highly relevant to psoriasis pathogenesis, including pattern-recognition receptors, cytokine receptors, and stress-responsive signaling modules. Engagement of receptors such as Toll-like receptor 7 (TLR7), interleukin-17 receptor (IL-17R), tumor necrosis factor receptor (TNFR), and interleukin-1 receptor (IL-1R) initiates signaling cascades that converge on shared adaptor proteins and kinase complexes. In addition to receptor activation itself, altered intracellular trafficking of TLR7 has been shown to aggravate psoriasis-like inflammation, suggesting that receptor localization and signal compartmentalization may also shape NF-κB-dependent inflammatory output (25–30). Central to this convergence are tumor necrosis factor receptor–associated factors (TRAFs), particularly TRAF3 and TRAF6, which function as molecular scaffolds linking receptor-proximal events to downstream kinase activation.

**Figure 1:**
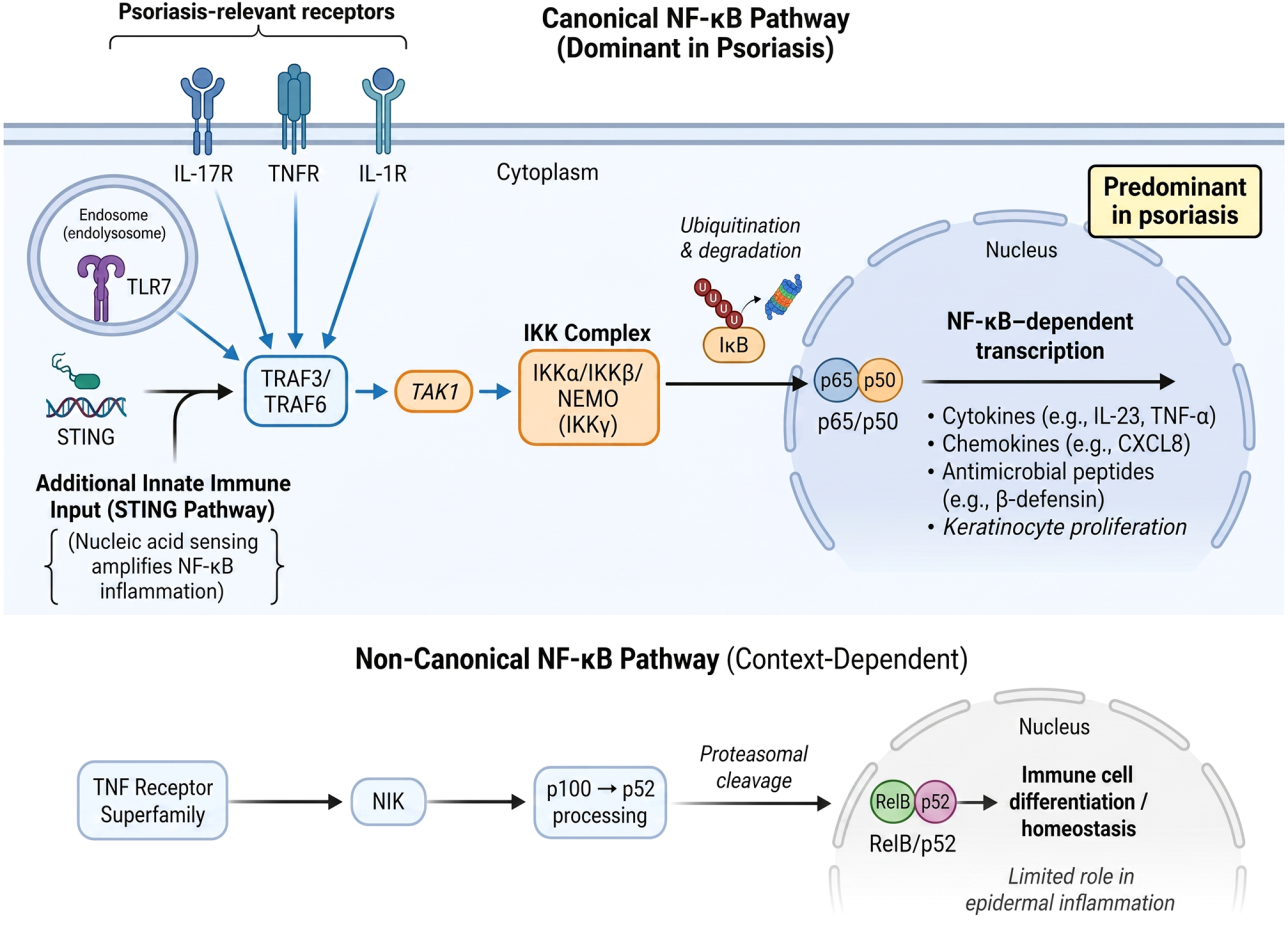
Canonical and non-canonical NF-κB signaling in psoriasis. Psoriasis-relevant receptors, including TLR7, IL-17R, TNFR, and IL-1R, converge on shared adaptor molecules (TRAF3/6), leading to TAK1 and IKK complex activation and canonical NF-κB–dependent transcription of inflammatory and keratinocyte survival genes. In contrast, non-canonical NF-κB signaling mediated by NIK-driven p100 processing plays a more restricted, context-dependent role in immune regulation.

A critical signaling node downstream of TRAFs is transforming growth factor-β–activated kinase 1 (TAK1), which integrates signals from multiple receptor systems (31, 32). Activated TAK1 phosphorylates the IκB kinase (IKK) complex, composed of catalytic subunits IKKα and IKKβ and the regulatory subunit NEMO (IKKγ) (33). This phosphorylation cascade leads to degradation of IκB inhibitors, allowing NF-κB dimers to translocate into the nucleus and initiate transcription of inflammatory genes. In the context of psoriasis, this canonical cascade drives the expression of cytokines, chemokines, antimicrobial peptides, and regulators of keratinocyte proliferation and survival. In contrast, the non-canonical NF-κB pathway, which relies on NF-κB–inducing kinase (NIK)–dependent processing of p100 to p52, is typically activated by a more restricted set of receptors, such as certain members of the TNF receptor superfamily (34–38). Although this pathway contributes to lymphoid organogenesis and immune cell differentiation, its role in psoriasis appears more limited and context dependent. Current evidence suggests that non-canonical signaling may modulate immune cell homeostasis rather than directly driving epidermal inflammation, highlighting the dominant role of canonical NF-κB activation in psoriatic lesions (**Figure 1**).

Beyond classical immune receptors, psoriasis-relevant NF-κB activation also arises from cytosolic nucleic acid sensing pathways, including the STING (stimulator of interferon genes) axis (39, 40). While STING signaling is traditionally associated with type I interferon responses, it also engages canonical NF-κB through TRAF-dependent mechanisms, thereby linking innate immune sensing of endogenous or microbial DNA to sustained inflammatory transcriptional programs in the skin. Together, these pathways underscore a fundamental principle in psoriasis biology: diverse upstream receptors funnel their signals through a limited set of shared molecular nodes—TRAFs, TAK1, and the IKK complex—before converging on NF-κB–dependent gene regulation. This architectural organization enables efficient signal integration but also creates vulnerability points for inflammatory amplification.

### 2.2 Subunit specificity and context-dependent NF-κB programs: c-Rel as a key inflammatory regulator

Although NF-κB is often discussed as a single signaling entity, its transcriptional activity is mediated by distinct combinations of subunits, including RelA (p65), RelB, c-Rel, p50, and p52. These subunits form various homo- or heterodimers with unique DNA-binding affinities and cofactor interactions, resulting in cell type–specific and stimulus-dependent transcriptional programs. Increasing evidence indicates that such subunit specificity is particularly relevant in chronic inflammatory diseases such as psoriasis. Among NF-κB family members, c-Rel has emerged as a critical regulator of inflammatory immunity. Unlike RelA, which is ubiquitously expressed and broadly involved in inflammatory gene induction, c-Rel expression is more tightly regulated and enriched in immune cells, including dendritic cells and lymphocytes (41, 42). This distribution allows c-Rel to selectively shape immune activation thresholds and cytokine production, particularly in contexts involving innate immune priming and adaptive immune reinforcement. Importantly, the emphasis on c-Rel should not be interpreted as indicating that c-Rel/p50 completely replaces the canonical p65/p50 complex in psoriasis. The p65/p50 dimer represents a broadly engaged canonical NF-κB module downstream of multiple psoriasis-relevant receptors, including TNFR, IL-1R, IL-17R, and TLRs. In contrast, c-Rel/p50 appears to contribute more selectively to immune-cell-associated transcriptional programs, particularly in the context of TLR7-driven inflammation. Thus, p65/p50 and c-Rel/p50 should be viewed as context-dependent and potentially co-existing NF-κB programs that differ in cellular distribution, upstream activation context, and transcriptional output.

**Figure 2:**
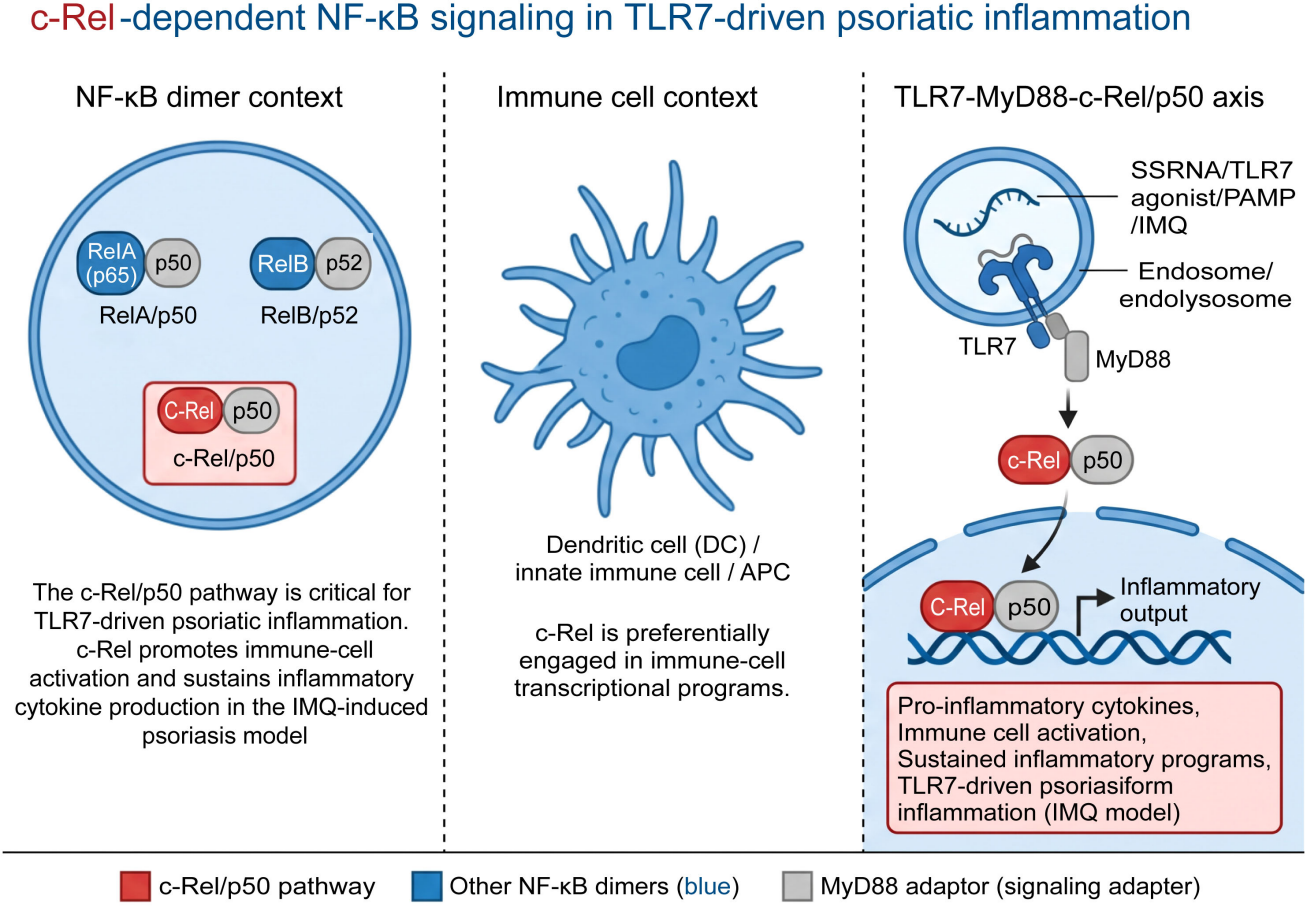
c-Rel–dependent NF-κB subunit specificity in psoriasis. NF-κB signaling operates as a modular transcriptional system in which distinct subunit combinations drive context-dependent inflammatory programs. In immune cells, c-Rel is preferentially engaged downstream of TLR7 activation and selectively promotes pro-inflammatory cytokine expression and immune activation in psoriasis. This subunit-specific regulation enables localized innate immune signals to generate sustained adaptive inflammatory responses while remaining distinct from ubiquitous RelA-mediated NF-κB activity.

Recent mechanistic studies have demonstrated that c-Rel plays an indispensable role in TLR7-driven psoriatic inflammation (43). TLR7 activation, a central feature of imiquimod-induced psoriasis models, preferentially engages c-Rel–dependent transcriptional programs that amplify inflammatory cytokine production and immune cell activation. Genetic or functional disruption of c-Rel markedly attenuates TLR7-induced skin inflammation, positioning c-Rel as a key executor that translates innate immune sensing into sustained inflammatory output. Importantly, the role of c-Rel extends beyond a simple amplification of canonical NF-κB signaling. By selectively regulating subsets of inflammatory genes, c-Rel contributes to the qualitative nature of immune responses, influencing cytokine profiles, immune cell recruitment, and the balance between inflammatory persistence and resolution. In psoriasis, this selective transcriptional control may help explain how localized innate immune triggers give rise to durable adaptive immune activation within the skin. These observations reinforce the concept that NF-κB functions not merely as a binary on–off switch, but as a modular transcriptional system whose output depends on subunit composition, upstream signaling context, and cellular identity. Recognizing this complexity is essential for understanding how NF-κB integrates innate and adaptive immune signals in psoriasis and for identifying strategies to selectively disrupt pathogenic inflammation while preserving essential immune functions (**Figure 2**).

## 3 Innate immune entry points converging on NF-κB in psoriasis

Innate immune activation represents a critical initiating event in psoriasis, translating environmental cues and endogenous danger signals into sustained inflammatory responses (44). Multiple innate immune sensors operate within the psoriatic skin microenvironment, yet a unifying feature of these pathways is their convergence on NF-κB signaling. This convergence enables heterogeneous upstream stimuli to be integrated into a coherent transcriptional program that fuels chronic inflammation and primes adaptive immune responses.

### 3.1 Nucleic acid sensing and STING–NF-κB–mediated inflammation

**Figure 3:**
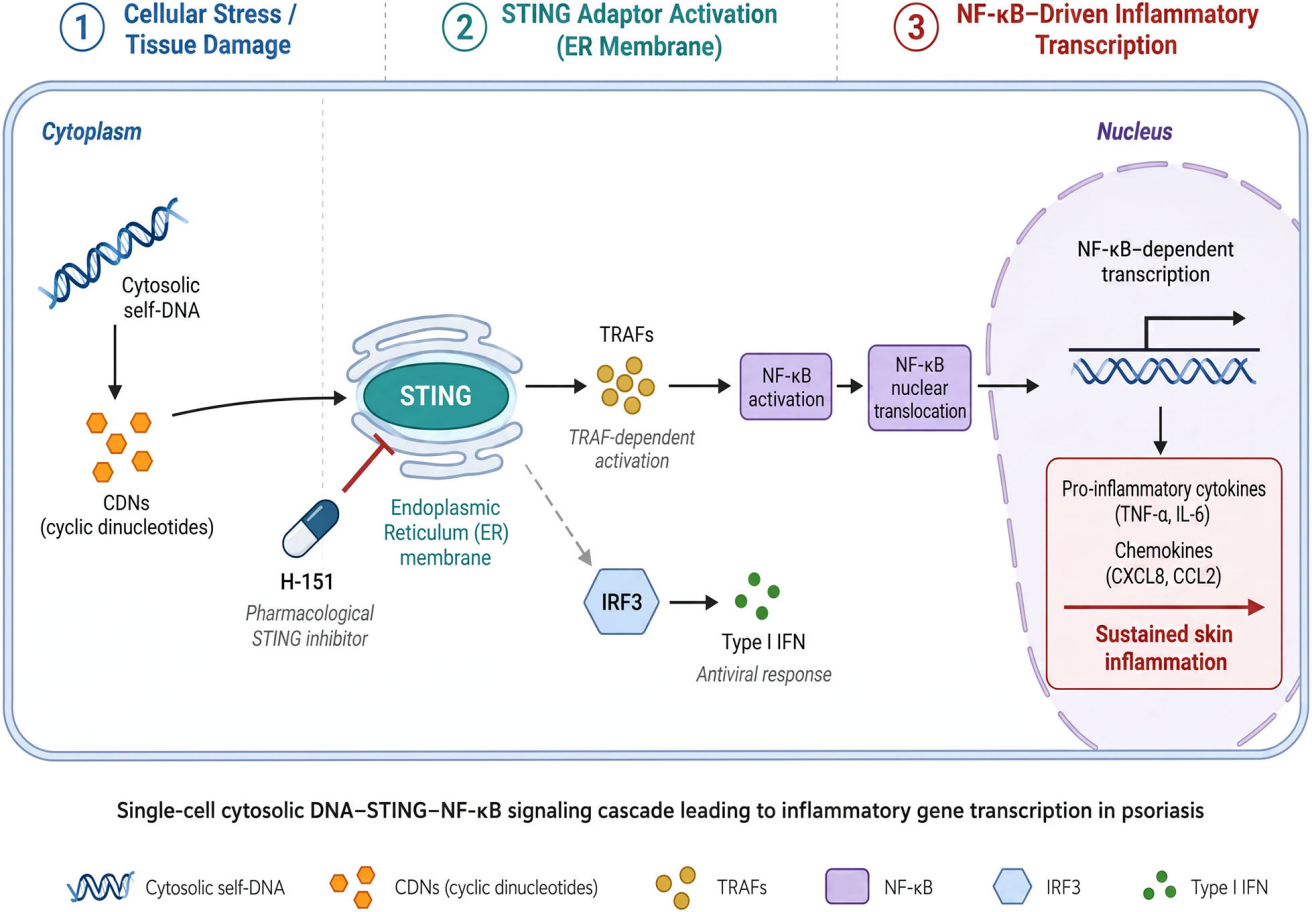
STING-mediated canonical NF-κB activation in psoriatic inflammation. Cytosolic DNA and cyclic dinucleotides activate STING in keratinocytes and innate immune cells. STING engages TRAF-dependent signaling to activate the canonical NF-κB pathway, with p65/p50 shown as the representative downstream dimer in this figure. This depiction is intended to illustrate STING-dependent canonical NF-κB activation and should be distinguished from the c-Rel/p50 module highlighted in TLR7-driven immune-cell responses. Pharmacological inhibition of STING by H-151 attenuates STING/NF-κB-mediated inflammation, supporting STING as an upstream innate immune entry point in psoriasis-like inflammation.

Cytosolic nucleic acid sensing has emerged as an important innate immune mechanism in inflammatory skin diseases. Among these pathways, the stimulator of interferon genes (STING) serves as a central adaptor that detects cytosolic DNA and cyclic dinucleotides generated during cellular stress, infection, or tissue damage (45, 46). While STING is classically associated with type I interferon production through IRF3 activation, accumulating evidence indicates that STING signaling also robustly engages the canonical NF-κB pathway (47). Upon activation, STING recruits downstream signaling molecules that facilitate canonical NF-κB activation via TRAF-dependent mechanisms, leading to the transcription of proinflammatory cytokines and chemokines. In this context, **Figure 3** primarily depicts STING–TRAF-dependent canonical NF-κB signaling in keratinocytes and innate immune cells, with p65/p50 used as the representative downstream NF-κB dimer. This should be distinguished from the c-Rel/p50 module highlighted in TLR7-driven immune-cell responses. In the context of psoriasis, this dual signaling capacity allows STING to function not only as an interferon-inducing sensor but also as a potent amplifier of inflammatory gene expression (48–52). Such amplification is particularly relevant in lesional skin, where repeated cellular stress and damage may sustain STING activation independently of overt infection. Pharmacological targeting of this pathway provides direct evidence for its pathogenic relevance. The STING antagonist H-151 has been shown to ameliorate psoriasis-like inflammation by suppressing STING-dependent NF-κB activation, resulting in reduced expression of inflammatory mediators and improved skin pathology (53). These findings position STING as a functional innate immune entry point that feeds directly into the NF-κB inflammatory hub, rather than acting solely through interferon-driven pathways (**Figure 3**).

Importantly, the ability of STING to activate NF-κB highlights a mechanism by which nucleic acid sensing can contribute to chronic inflammation without relying exclusively on antiviral immune programs. This distinction is particularly relevant for psoriasis, where excessive type I interferon signaling alone cannot fully account for disease persistence. Instead, STING-mediated NF-κB activation provides a sustained transcriptional output that reinforces local inflammation and creates a permissive environment for downstream adaptive immune activation.

### 3.2 The TLR7–NF-κB/c-Rel axis as an innate-to-adaptive and epidermal amplification bridge

Toll-like receptors (TLRs) represent another major class of innate immune sensors implicated in psoriasis (54, 55). Among them, TLR7 has been extensively studied because topical imiquimod (IMQ), a TLR7/8 agonist, induces psoriasis-like skin inflammation in mice. The IMQ-induced model is a foundational experimental system that recapitulates key clinical and histopathological features of psoriasis and is largely mediated through the IL-23/IL-17 axis (56–58). However, the IMQ-induced model remains an acute experimental mouse model and cannot fully substitute for human psoriasis studies. Its translational relevance has been debated because it does not completely capture the chronicity, genetic heterogeneity, disease recurrence, comorbidity profile, or treatment-response diversity of human psoriasis. Therefore, findings derived from IMQ-based models should be interpreted with caution and further validated using patient-derived samples, human skin models, and clinical datasets. Activation of TLR7 in dendritic cells and other innate immune populations initiates a cascade of inflammatory signaling events that converge on NF-κB. Activation of TLR7 in dendritic cells, plasmacytoid dendritic cells, and other innate immune populations initiates inflammatory signaling cascades that converge on NF-κB-dependent transcriptional programs. Recent studies have further refined this model by distinguishing TLR7-dependent inflammatory signaling from formulation- or vehicle-associated effects in IMQ/Aldara-based models (59). These findings support the central contribution of TLR7 signaling to IMQ-driven inflammation, while also emphasizing that conclusions drawn from these models should be interpreted within their specific experimental context.

Importantly, TLR7-driven inflammation is not determined solely by ligand recognition. Aberrant intracellular trafficking of TLR7 has recently emerged as an additional regulatory layer in psoriasis-like inflammation. Zhu et al. showed that dysregulated TLR7 trafficking aggravates psoriatic inflammation and identified SHP2 as a regulatory node controlling this process (30). Pharmacological allosteric inhibition of SHP2 altered TLR7 trafficking and reduced downstream inflammatory responses, thereby linking receptor localization dynamics to TLR7–NF-κB activation. This study provides mechanistic evidence that spatial regulation of TLR7 can shape the intensity and persistence of innate immune signaling, rather than merely acting as a passive upstream trigger. Together with recent work on TLR7 antagonism, these findings indicate that receptor-level regulation of the TLR7 pathway may represent a more selective strategy for attenuating upstream innate immune entry into NF-κB activation than broad downstream NF-κB inhibition (60).

Recent evidence highlights the importance of c-Rel, a distinct NF-κB subunit, in mediating TLR7-driven inflammation (43). Unlike the broadly acting RelA subunit, c-Rel selectively regulates transcriptional programs associated with immune activation and cytokine production (42). In TLR7-stimulated settings, c-Rel is preferentially engaged to drive the expression of inflammatory mediators that promote immune cell activation and recruitment. Functional studies demonstrate that c-Rel is indispensable for TLR7-induced psoriatic inflammation, as disruption of c-Rel signaling markedly attenuates skin inflammation in IMQ models (43). In TLR7-driven psoriatic inflammation, NF-κB activation should therefore be interpreted as a subunit-dependent transcriptional process rather than a uniform pathway (43). The broadly engaged RelA/p65–p50 dimer contributes to canonical NF-κB inflammatory signaling in multiple cell types, whereas c-Rel/p50 appears to be more selectively associated with immune-cell transcriptional programs (43). In dendritic cells and other innate immune cells, endosomal TLR7 activation engages MyD88-dependent signaling and can preferentially promote c-Rel-dependent inflammatory outputs. Importantly, this does not imply that c-Rel/p50 replaces p65/p50. Rather, these dimers may coexist downstream of TLR7 activation, with their relative contributions depending on cell type, stimulation context, signal duration, and target-gene regulatory architecture. This subunit-specific organization helps explain how TLR7 activation generates immune-cell-biased inflammatory programs that support cytokine production, immune-cell activation, and downstream Th17-associated inflammation.

These findings underscore the role of c-Rel as a molecular bridge that links innate immune sensing to the downstream activation of adaptive immune pathways. By shaping cytokine profiles and costimulatory signals in antigen-presenting cells, c-Rel-dependent NF-κB activity facilitates the transition from acute innate responses to sustained adaptive immune engagement.

In addition to myeloid and dendritic cell compartments, TLR7–NF-κB signaling has also been described in keratinocytes, suggesting that this axis may contribute to psoriasis through both immune-cell-intrinsic and epidermal-intrinsic mechanisms. Previous work showed that TLR7 expression can be induced in differentiated keratinocytes and that IMQ stimulation promotes IκBα phosphorylation, NF-κB transcriptional activation, and inflammatory mediator production, including TNF-α and IL-8 (61). Importantly, knockdown of TLR7 reduced IMQ-induced NF-κB activity and cytokine production, supporting a direct role for keratinocyte TLR7 in epidermal inflammatory amplification. This finding complements dendritic cell–centered studies by indicating that TLR7 signaling is not restricted to innate immune cells but may also operate within keratinocytes to reinforce local cytokine and chemokine output.

**Figure 4:**
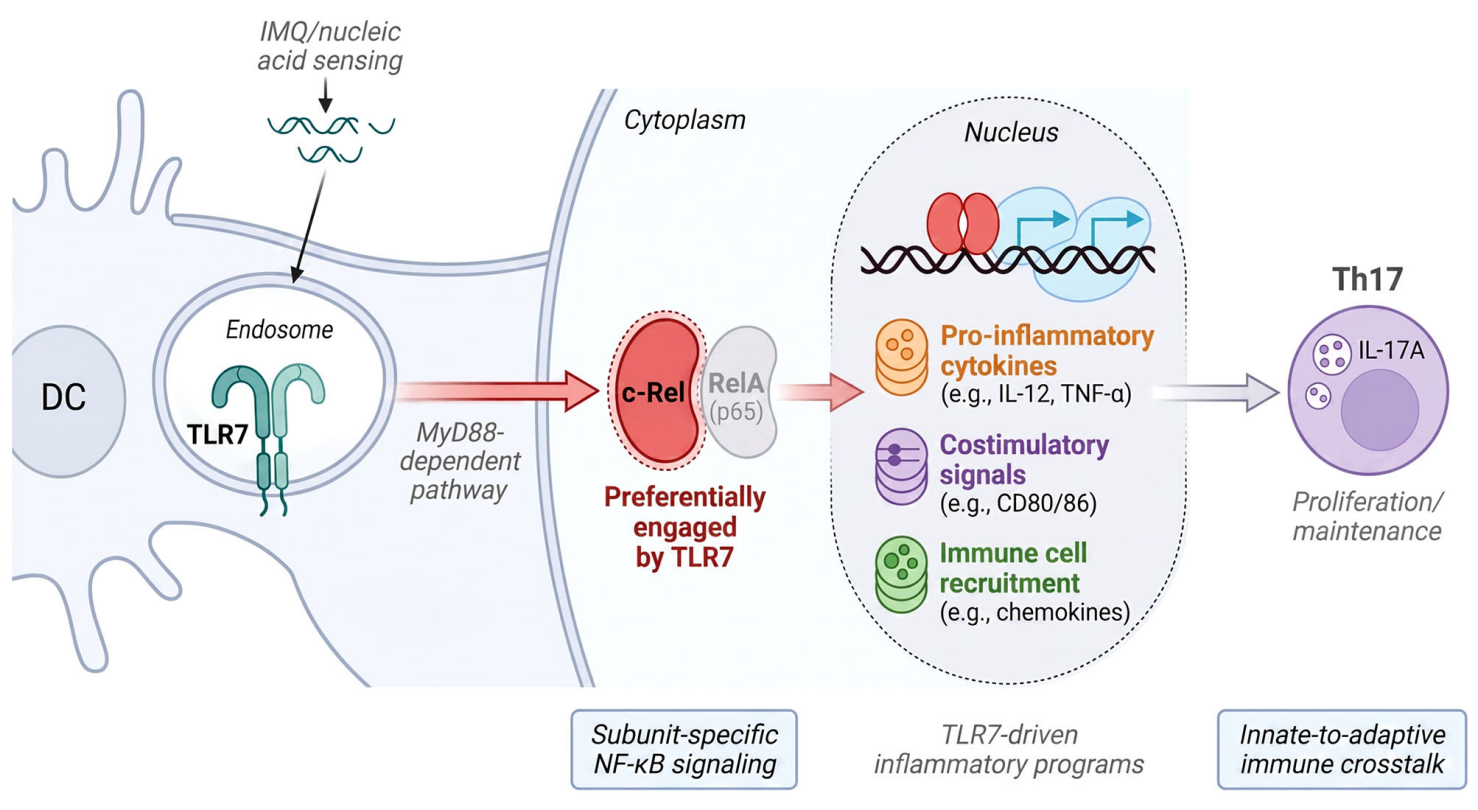
TLR7–NF-κB subunit usage in dendritic cells during psoriasis-like inflammation. TLR7 activation in dendritic cells can engage multiple canonical NF-κB dimers, including p65/p50 and c-Rel/p50. The figure highlights c-Rel/p50 because recent evidence indicates that c-Rel is particularly important for full TLR7-driven inflammatory responses in IMQ-induced psoriasis-like models. This should not be interpreted as an all-or-nothing replacement of p65/p50. Rather, p65/p50 and c-Rel/p50 may coexist within the same cell and contribute to overlapping but non-identical transcriptional programs. The relative contribution of c-Rel/p50 may depend on cell type, stimulation context, signal duration, and promoter/enhancer architecture of target inflammatory genes.

Taken together, the TLR7–NF-κB/c-Rel axis exemplifies how NF-κB subunit specificity and receptor-level regulation confer functional directionality within innate immune signaling. Rather than indiscriminately activating inflammatory genes, TLR7-associated pathways may promote psoriasis-like inflammation through multiple regulatory layers: intracellular receptor trafficking, c-Rel-dependent transcription in immune cells, and NF-κB-dependent cytokine production in keratinocytes. These interconnected layers provide a mechanistic basis for the transition from innate immune activation to Th17 cell expansion and long-term inflammatory maintenance in psoriatic skin (**Figure 4**). However, because much of the evidence for TLR7-dependent inflammation derives from IMQ/Aldara-induced models, the extent to which TLR7 trafficking and keratinocyte-intrinsic TLR7 signaling contribute to different clinical subtypes or stages of human psoriasis remains to be further clarified.

### 3.3 NF-κB–inflammasome crosstalk in psoriasis: from priming to pyroptosis

Beyond pattern-recognition receptors and nucleic acid sensors, innate immune signaling in psoriasis is further shaped by inflammasome pathways. Inflammasomes are cytosolic multiprotein platforms that sense microbial, stress-associated, or damage-associated signals and promote inflammatory caspase activation. In psoriasis, several inflammasome sensors have been implicated, including NLRP3, NLRP1, and AIM2, although the strength of evidence differs among them (62, 63). NLRP3 remains the most extensively studied inflammasome in psoriatic inflammation, whereas NLRP1 and AIM2 provide additional routes through which epidermal stress, barrier disruption, and cytosolic nucleic acids may be converted into inflammatory cytokine maturation (64, 65).

Mechanistically, NF-κB intersects with inflammasome signaling most prominently at the priming stage. Canonical NF-κB activation downstream of TLRs, IL-1R, TNFR, and other inflammatory receptors induces the transcription of inflammasome-related components and substrates, including NLRP3, pro–IL-1β, and pro–IL-18 (62, 63). This priming step prepares keratinocytes and innate immune cells for subsequent inflammasome activation. A second signal, such as potassium efflux, mitochondrial dysfunction, reactive oxygen species, lysosomal damage, or cytosolic nucleic acids, can then promote inflammasome assembly. In the canonical inflammasome pathway, sensor proteins such as NLRP3, NLRP1, or AIM2 recruit ASC and caspase-1, resulting in caspase-1 activation, maturation of IL-1β and IL-18, cleavage of gasdermin D, and pyroptotic cell death. This mechanism is particularly relevant to psoriasis because IL-1 family cytokines and keratinocyte-derived danger signals can reinforce epidermal inflammation and promote downstream Th17-associated immune responses (23). Thus, NF-κB does not merely operate in parallel with inflammasomes; rather, it establishes the transcriptional competence required for inflammasome responsiveness. Once activated, inflammasomes can feed back into the NF-κB-centered inflammatory network through IL-1β/IL-1R signaling, IL-18-mediated immune activation, and pyroptosis-associated release of damage-associated molecular patterns (24, 63, 66). These outputs further stimulate NF-κB activity in neighboring keratinocytes and immune cells, creating a self-amplifying inflammatory loop within psoriatic lesions. In addition to canonical inflammasomes, non-canonical inflammasome pathways may also contribute to psoriatic inflammation. Non-canonical inflammasome activation involves inflammatory caspases, such as caspase-4/5 in humans and caspase-11 in mice, which can directly cleave gasdermin D and induce pyroptosis (67, 68). Gasdermin-mediated membrane pore formation promotes cytokine release and cellular damage, potentially amplifying local inflammation. Although the relative contribution of non-canonical inflammasomes to human psoriasis remains less clearly defined than that of NLRP3, the involvement of gasdermin cleavage and pyroptotic signaling provides a mechanistic link between innate immune sensing, epidermal injury, and NF-κB-dependent inflammatory amplification (68, 69).

**Figure 5:**
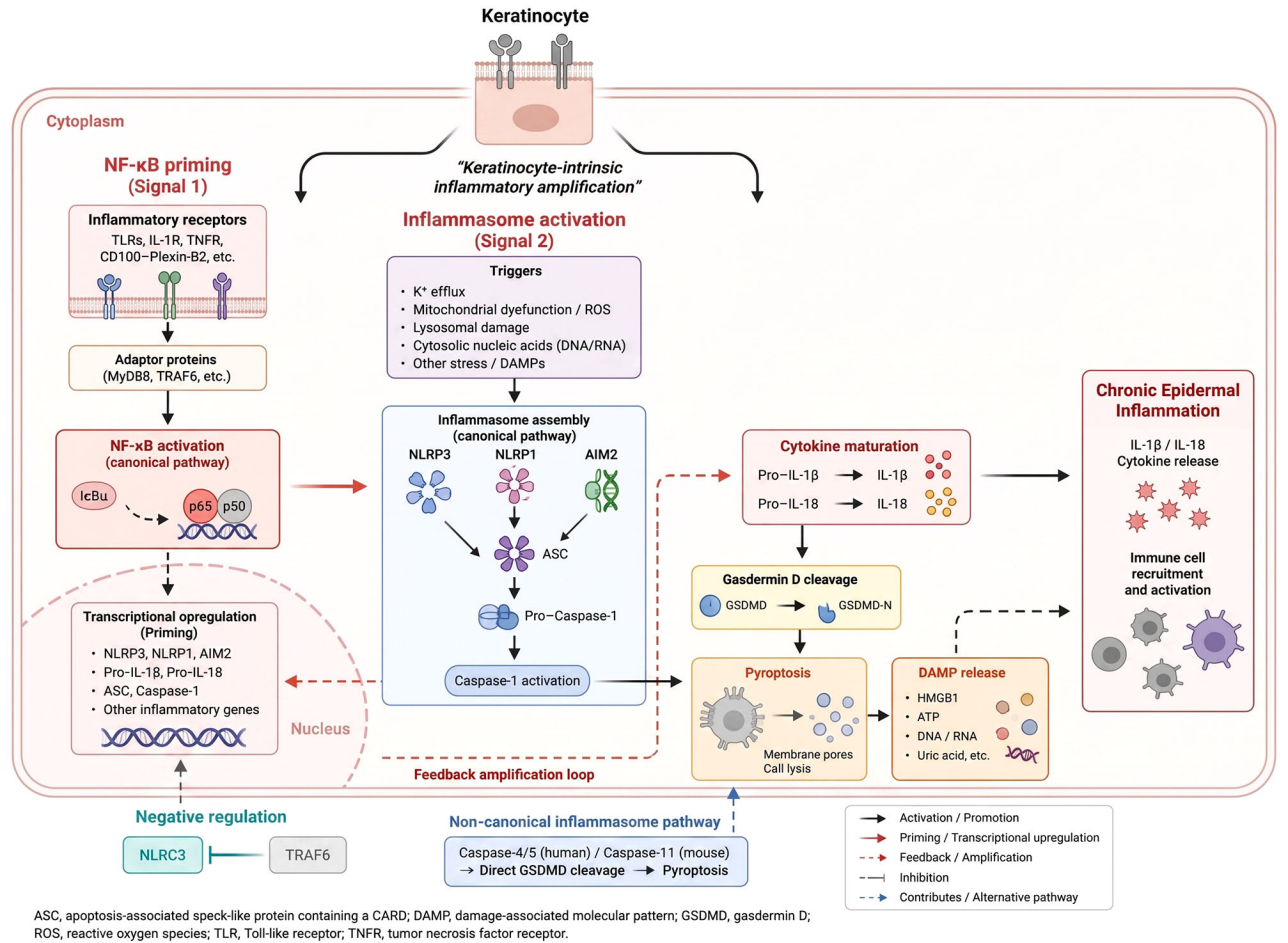
Bidirectional crosstalk between NF-κB and inflammasome pathways in psoriasis. NF-κB and inflammasome pathways form a bidirectional regulatory loop that amplifies epidermal inflammation in psoriasis. NF-κB provides a priming signal by inducing inflammasome components and pro–IL-1β, whereas inflammasome activation feeds back to reinforce NF-κB signaling. Keratinocyte-intrinsic CD100–Plexin-B2 promotes this coupling, while NLRC3 restrains excessive inflammation by limiting TRAF6-dependent NF-κB activation.

Keratinocytes are central participants in this NF-κB–inflammasome loop. For example, the CD100–Plexin-B2 axis has been shown to activate both NF-κB and inflammasome pathways in keratinocytes, thereby promoting inflammatory responses in psoriasis-like models. Conversely, regulatory NLR family members can restrain excessive inflammation by interacting with NF-κB-related signaling components. NLRC3, for instance, modulates NF-κB-mediated inflammatory responses through its interaction with TRAF6, limiting TRAF6-dependent signaling and inflammatory escalation. These examples illustrate that inflammasome pathways are not isolated inflammatory modules but are embedded within NF-κB-centered signaling networks (**Figure 5**).

Collectively, the NF-κB–inflammasome interface provides a mechanistic explanation for how transient danger signals can be converted into sustained inflammatory amplification in psoriasis. NF-κB contributes primarily by licensing inflammasome priming, while inflammasome activation, IL-1β/IL-18 maturation, gasdermin cleavage, and pyroptosis feed back to reinforce NF-κB-dependent cytokine and chemokine production. However, the relative importance of NLRP3, NLRP1, AIM2, and non-canonical inflammasome pathways likely varies across cell types, disease stages, and experimental models. Future studies using patient-derived skin samples, single-cell profiling, and spatial inflammatory mapping will be required to define which inflammasome modules are most relevant to specific psoriasis phenotypes. **Table 1** delineates major innate immune entry points that converge on NF-κB signaling, highlighting shared adaptor nodes and inflammatory outputs across distinct sensing pathways.

**Table 1:** Innate immune entry points converging on NF-κB in psoriasis.

Innate sensor/pathway	Cell type	Key NF-κB adaptor or node	Downstream inflammatory output	Representative study	Representative PMID(s)
STING (cytosolic DNA sensing)	Keratinocytes, immune cells	TRAFs → canonical NF-κB	Pro-inflammatory cytokines, chemokines	H-151 STING antagonist study	PMID: 34460100
TLR7 (IMQ-induced)	Dendritic cells	c-Rel–dependent NF-κB	Innate immune activation, Th17 priming	TLR7–c-Rel psoriasis study	PMID: 39586195
CD100–Plexin-B2	Keratinocytes	NF-κB + inflammasome	IL-1 family cytokines, epidermal inflammation	CD100–Plexin-B2 JID	PMID: 28927892
Inflammasome (priming phase)	Keratinocytes	NF-κB priming	Pro-IL-1β transcription	Inflammasome crosstalk studies	PMID: 34072753; PMID: 32896537
NLRC3 (regulatory NLR)	Keratinocytes/immune cells	TRAF6 inhibition	Dampened NF-κB signaling	NLRC3–TRAF6 study	PMID: 39615555

## 4 Adaptive immune maintenance: Th17/IL-17 signaling converging on NF-κB

While innate immune activation initiates psoriatic inflammation, adaptive immune responses are essential for its persistence and chronicity. Among adaptive immune subsets, Th17 cells and their signature cytokines, particularly IL-17A, play a dominant role in maintaining inflammatory circuits within the skin. A defining feature of this process is the convergence of Th17-associated signaling on NF-κB, which integrates immune-derived cues with tissue-resident responses to sustain disease activity (11, 12, 14). **Table 2** integrates adaptive immune maintenance with keratinocyte-intrinsic signal amplification, emphasizing how Th17-derived inputs are reinforced at the tissue level.

**Table 2:** Adaptive immune maintenance and keratinocyte signal amplification through NF-κB.

Axis/module	Primary cell type	NF-κB role	Key amplification mechanism	Functional consequence	Representative PMID(s)
APC → Th17 differentiation	Dendritic cells	Cytokine transcription	IL-23/IL-6/IL-1β production	Th17 lineage commitment	PMID: 19380832; PMID: 37098777
CYSLTR1–NF-κB axis	Immune cells	NF-κB modulation	Lipid mediator signaling	Enhanced Th17 differentiation	PMID: 38617532
IL-17R signaling	Keratinocytes	NF-κB & MAPK activation	TRAF-dependent signaling	Inflammatory gene expression	PMID: 27576147; PMID: 29938836
FXYD3–TRAF3 module	Keratinocytes	Signal gain control	Competitive TRAF3 binding	Amplified IL-17 responsiveness	PMID: 36693922
CCN1–NF-κB–IL-36 axis	Keratinocytes	Secondary cytokine induction	AKT/NF-κB + ERK/C/EBPβ	Immune recruitment & feedback	PMID: 36376243; PMID: 26099024

### 4.1 Th17 differentiation and NF-κB-dependent inflammatory reinforcement

**Figure 6:**
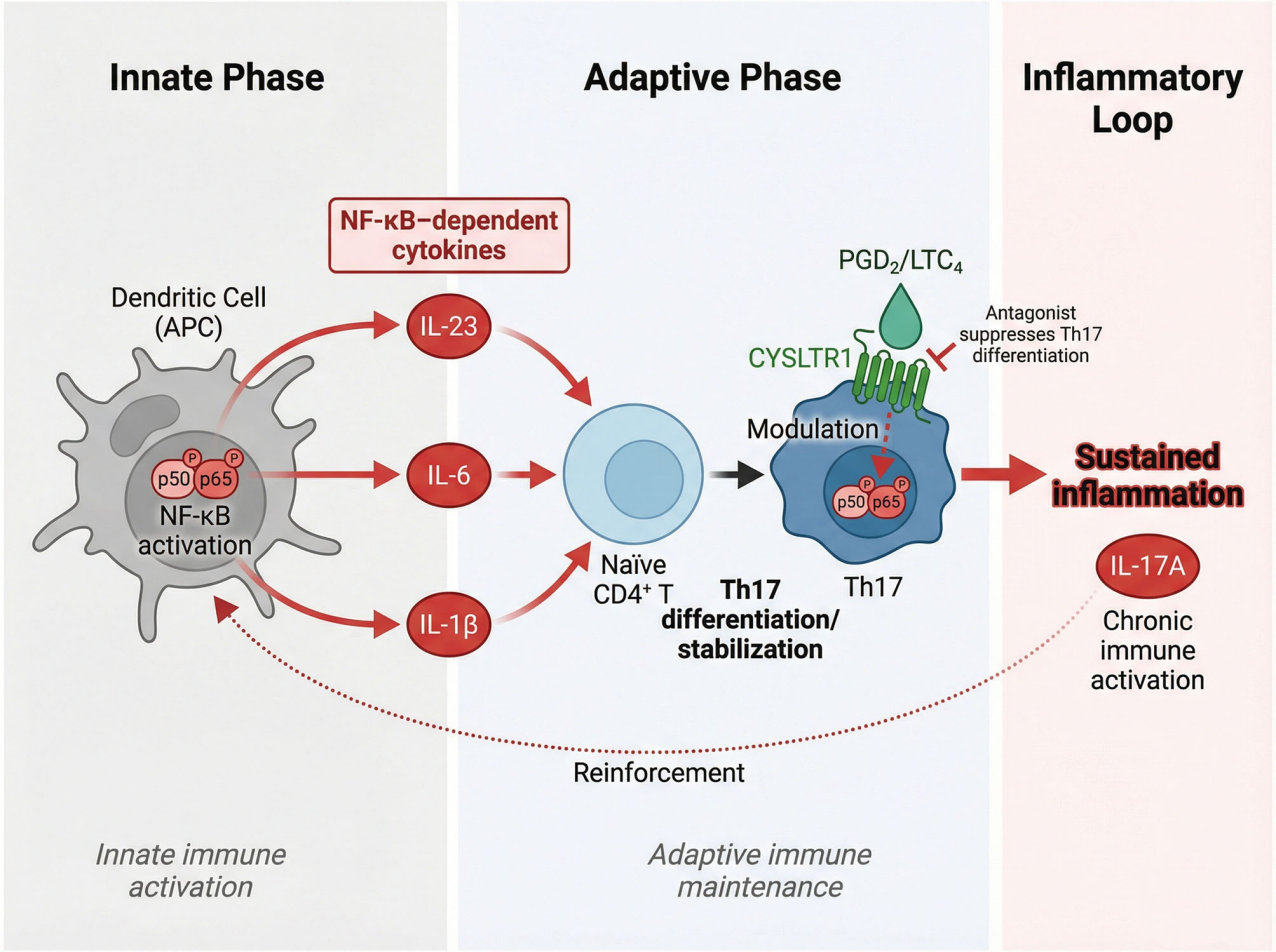
NF-κB–dependent reinforcement of Th17 differentiation in psoriasis. NF-κB signaling sustains Th17 differentiation and maintenance by coordinating cytokine production in antigen-presenting cells and integrating lipid mediator–dependent cues. NF-κB–driven expression of IL-23, IL-6, and IL-1β promotes Th17 lineage stabilization, while CYSLTR1-mediated lipid signaling further modulates NF-κB activity. This integrated signaling network reinforces adaptive immune inflammation and contributes to chronic disease persistence in psoriasis.

The differentiation and maintenance of Th17 cells require the coordinated action of multiple cytokines, costimulatory signals, and transcriptional regulators. Antigen-presenting cells activated during the innate phase produce cytokines such as IL-23, IL-6, and IL-1β, which promote Th17 lineage commitment and stabilization (70–73). NF-κB signaling plays a central role in this process by regulating cytokine production, costimulatory molecule expression, and inflammatory gene transcription in antigen-presenting cells. Beyond classical cytokine-driven pathways, emerging evidence highlights the contribution of lipid mediator–dependent signaling to Th17 regulation. Cysteinyl leukotriene receptor 1 (CYSLTR1), traditionally associated with allergic inflammation, has recently been implicated in psoriasis pathogenesis. Pharmacological antagonism of CYSLTR1 suppresses Th17 cell differentiation by modulating NF-κB signaling, revealing a previously underappreciated axis linking lipid mediators to adaptive immune responses (74). This lipid mediator–Th17–NF-κB axis expands the conceptual framework of Th17 regulation in psoriasis. Rather than acting solely downstream of cytokine receptors, NF-κB integrates signals from diverse immunological inputs, including lipid-derived inflammatory cues. Such integration reinforces Th17-driven inflammation and contributes to the robustness of adaptive immune maintenance within psoriatic lesions. Collectively, these findings underscore NF-κB as a key molecular node that sustains Th17 responses by coordinating inflammatory reinforcement across innate and adaptive immune compartments (**Figure 6**).

### 4.2 Keratinocyte IL-17 response: signal gain control at TRAF nodes

**Figure 7:**
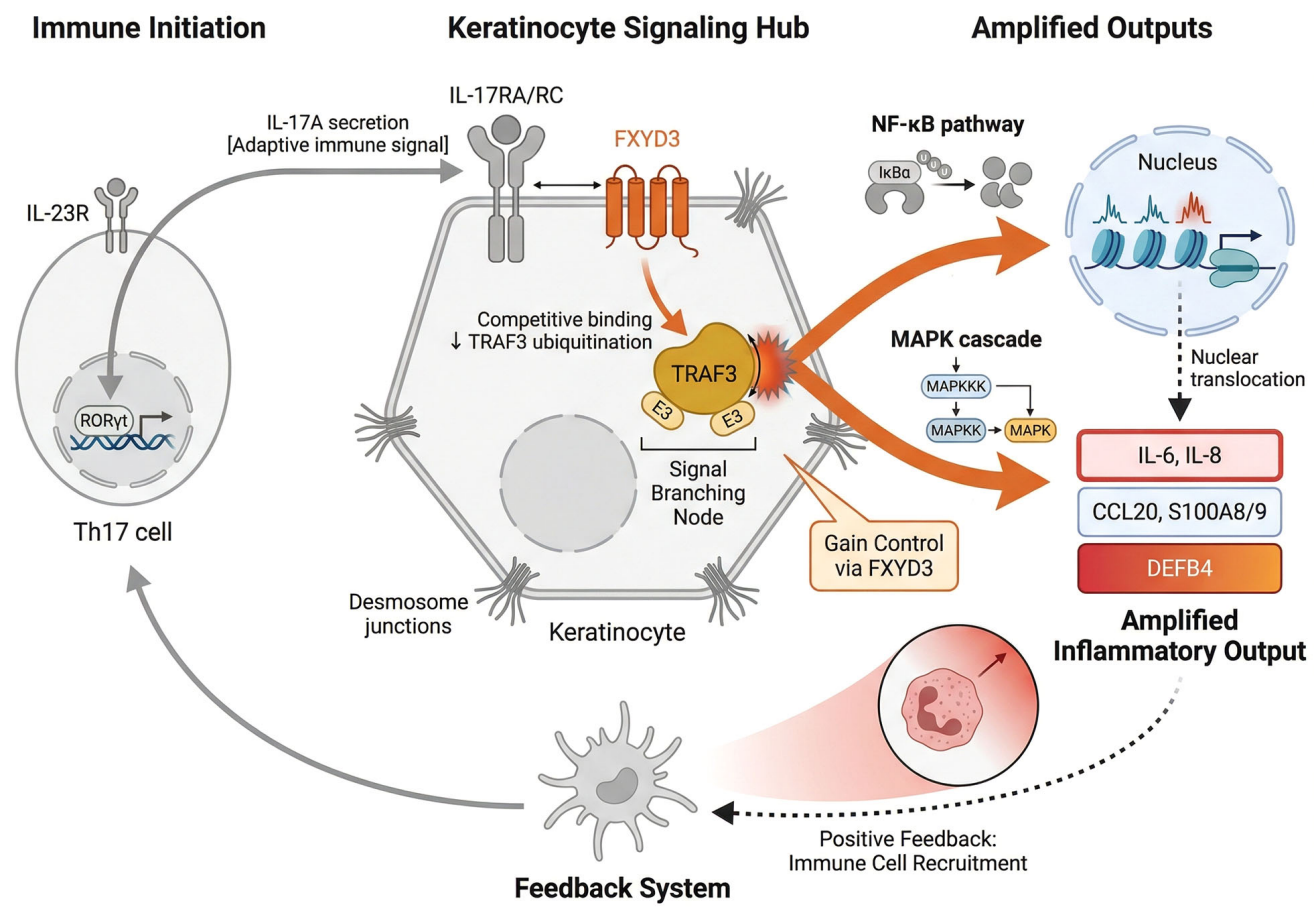
FXYD3-mediated signal gain control amplifies IL-17 responses in keratinocytes. IL-17A derived from Th17 cells activates IL-17 receptor signaling in keratinocytes, where TRAF adaptor proteins function as key signal branching nodes. FXYD3 competitively binds TRAF3, increasing signal gain toward NF-κB and MAPK activation. This keratinocyte-intrinsic amplification enhances inflammatory cytokine and chemokine production, establishing a positive feedback loop that reinforces Th17-driven chronic inflammation in psoriasis.

A hallmark of psoriasis is the exaggerated responsiveness of keratinocytes to IL-17A. Although IL-17A is produced predominantly by Th17 cells, its pathogenic impact depends critically on how keratinocytes interpret and amplify IL-17 signals. Recent studies have uncovered a sophisticated mechanism of signal gain control within keratinocytes that determines the intensity and duration of IL-17-induced inflammatory responses. Central to this mechanism is the regulation of TRAF adaptor proteins, which function as molecular branching points downstream of the IL-17 receptor (66, 75–77). TRAF proteins act as signaling “valves, “ directing receptor-derived signals toward distinct downstream pathways, including NF-κB and MAPK cascades. Fine-tuning at this level allows keratinocytes to calibrate inflammatory output in response to immune-derived stimuli. The transmembrane protein FXYD3 has emerged as a critical modulator of this process. FXYD3 enhances IL-17A signaling by competitively binding to TRAF3, thereby altering the composition of the IL-17 receptor signaling complex (78). Through this competitive interaction, FXYD3 shifts signaling dynamics in favor of sustained NF-κB and MAPK activation, leading to amplified expression of proinflammatory cytokines and chemokines in keratinocytes. This mechanism provides a compelling example of how keratinocyte-intrinsic factors actively shape immune signaling. Rather than serving as passive targets of Th17-derived cytokines, keratinocytes employ molecular regulators such as FXYD3 to increase signal gain at TRAF nodes, effectively amplifying adaptive immune inputs. Importantly, this amplification feeds back to the immune compartment by enhancing the production of chemotactic and inflammatory mediators that recruit and activate additional immune cells (**Figure 7**).

Thus, the FXYD3–TRAF3–NF-κB axis exemplifies a positive feedback loop between Th17 cells and keratinocytes, in which adaptive immune signals are reinforced at the tissue level to sustain chronic inflammation.

### 4.3 IL-36 and epidermal cytokine amplification through NF-κB

**Figure 8:**
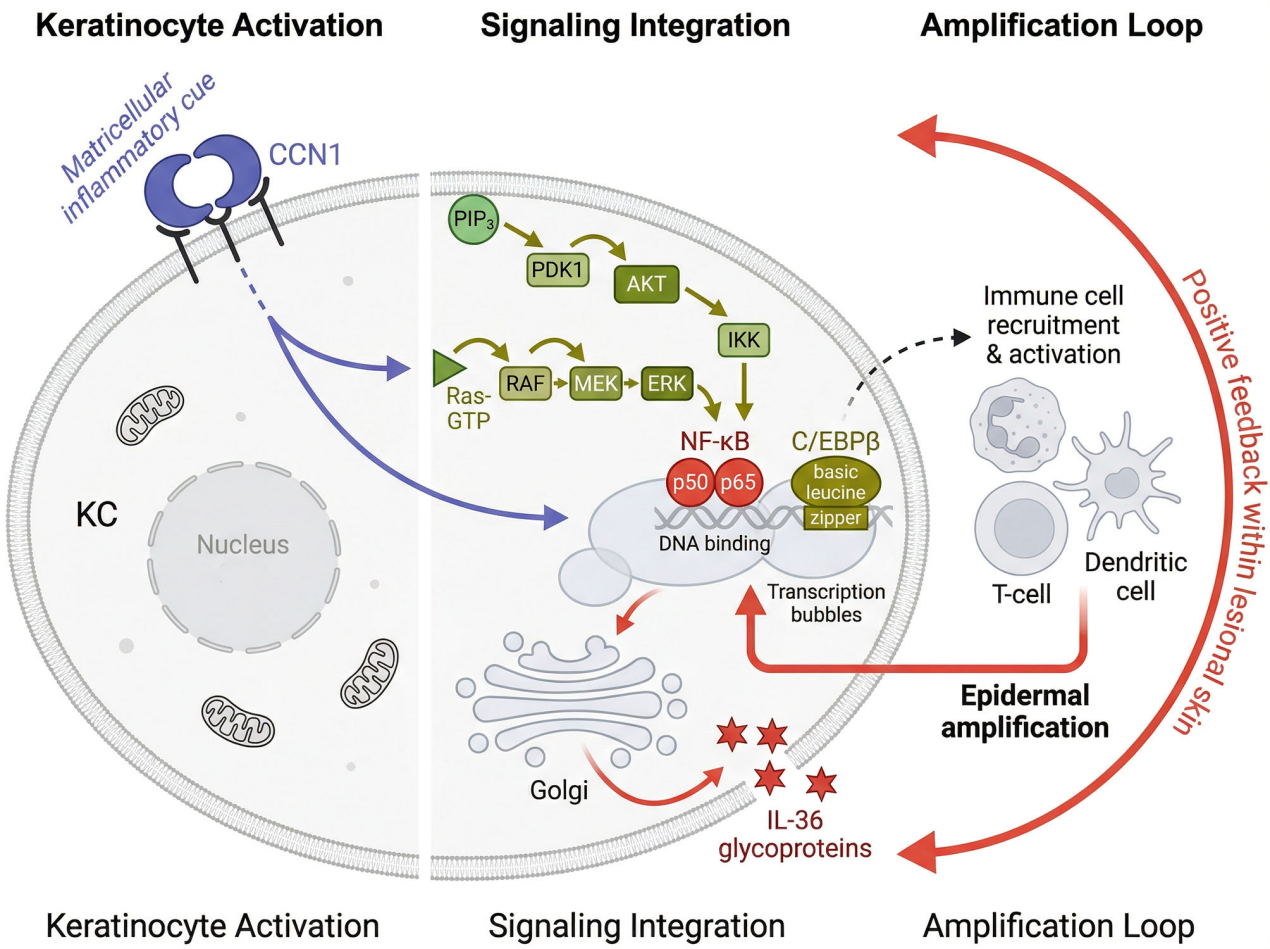
NF-κB–dependent IL-36 production amplifies epidermal inflammation in psoriasis. Keratinocytes actively amplify psoriatic inflammation by producing IL-36 family cytokines. CCN1 activates NF-κB–centered transcriptional programs through coordinated AKT and ERK/C/EBPβ signaling, inducing IL-36 expression. Keratinocyte-derived IL-36 enhances immune cell recruitment and activation and reinforces epidermal inflammatory loops, thereby sustaining localized immune–epidermal crosstalk and chronic inflammation in psoriatic lesions.

In addition to responding to immune-derived cytokines, keratinocytes actively contribute to adaptive immune maintenance by producing secondary inflammatory mediators that further shape the immune microenvironment. Among these, IL-36 family cytokines have gained increasing attention as potent amplifiers of psoriatic inflammation. IL-36 cytokines, produced predominantly by keratinocytes, act on both immune cells and epithelial cells to promote cytokine release, immune cell recruitment, and tissue inflammation (79, 80). NF-κB signaling plays a central role in regulating IL-36 expression, positioning it as a critical link between keratinocyte activation and immune amplification. Recent evidence demonstrates that CCN1, a matricellular protein involved in tissue remodeling and inflammation, upregulates IL-36 expression through coordinated activation of AKT/NF-κB and ERK/CEBPβ signaling pathways (81–83). This dual-pathway regulation highlights the integration of growth factor–associated signaling with inflammatory transcriptional programs in keratinocytes. By inducing IL-36 production, CCN1-mediated NF-κB activation amplifies epidermal cytokine output, creating a proinflammatory milieu that enhances immune cell infiltration and activation (84). This mechanism further reinforces the bidirectional communication between keratinocytes and immune cells, contributing to the persistence and spatial confinement of psoriatic lesions. Together, these findings illustrate how adaptive immune maintenance in psoriasis converges on NF-κB at multiple levels. Th17 differentiation and function, keratinocyte IL-17 signal amplification, and secondary epidermal cytokine production are all orchestrated through NF-κB-centered regulatory networks. By integrating immune-derived signals with keratinocyte-intrinsic amplification mechanisms, NF-κB ensures the durability and self-sustaining nature of psoriatic inflammation (**Figure 8**).

## 5 Keratinocyte-intrinsic NF-κB programs: hyperproliferation, barrier disruption, and inflammatory output

Keratinocytes are central effectors in psoriasis, yet their role extends far beyond that of passive targets responding to immune-derived cytokines. Increasing evidence indicates that keratinocytes harbor intrinsic NF-κB–dependent programs that actively drive epidermal hyperproliferation, barrier dysfunction, and inflammatory amplification. Through these programs, keratinocytes function as autonomous regulators of disease persistence and severity.

### 5.1 NF-κB-driven keratinocyte hyperproliferation and inflammatory loops

Epidermal hyperproliferation is a defining histopathological feature of psoriasis and contributes directly to plaque formation and barrier impairment. While immune cytokines initiate keratinocyte activation, sustained hyperproliferation is maintained by keratinocyte-intrinsic signaling pathways, among which NF-κB plays a pivotal role.

**Figure 9:**
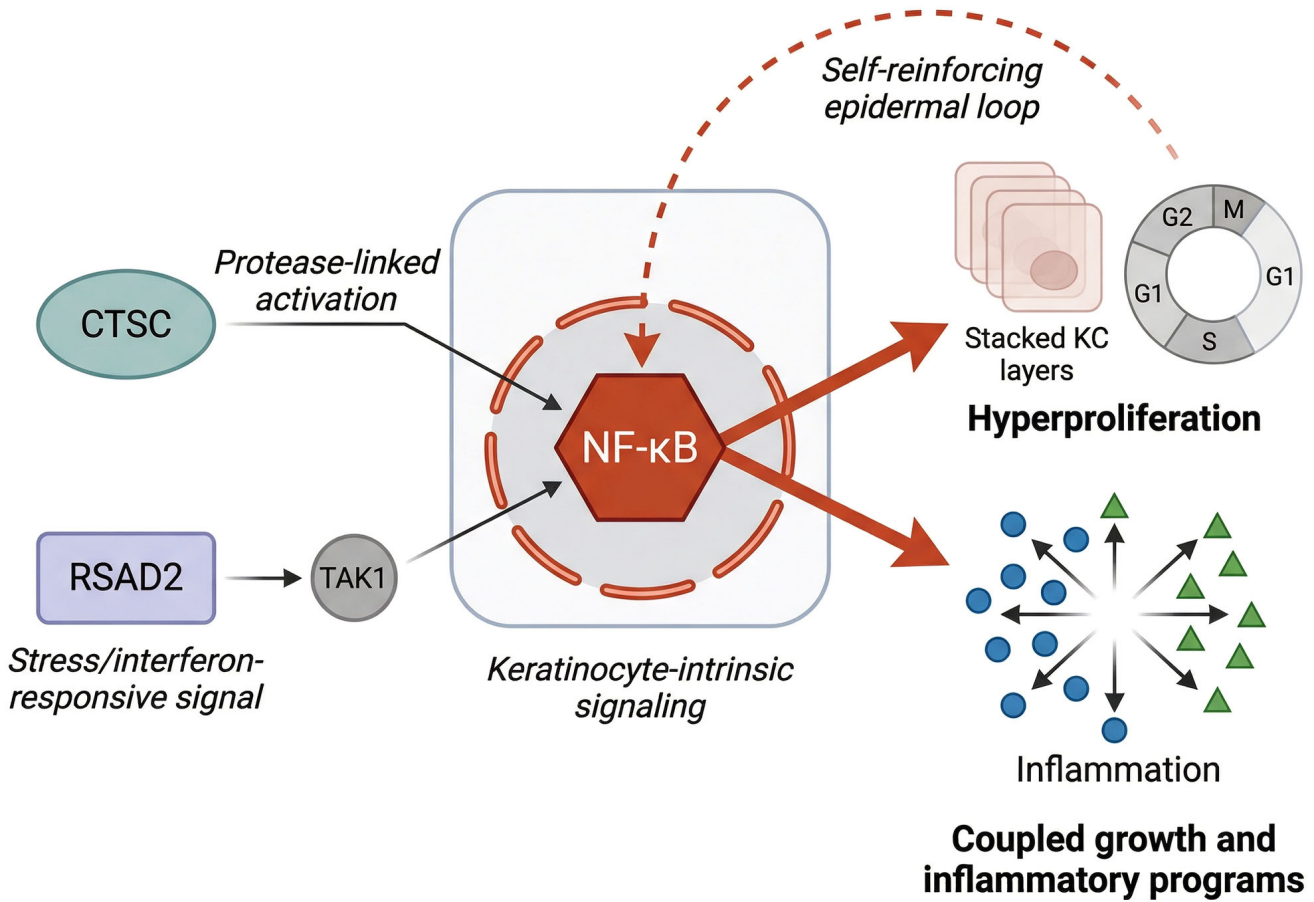
NF-κB–driven keratinocyte hyperproliferation and inflammatory self-amplification in psoriasis. Keratinocyte-intrinsic NF-κB signaling couples epidermal hyperproliferation with inflammatory mediator production in psoriasis. Dysregulated CTSC and stress-responsive RSAD2 converge on the TAK1/NF-κB axis, driving coordinated programs of cell cycle progression, survival, and inflammation. This integration establishes self-sustaining epidermal feedback loops that promote plaque formation, barrier dysfunction, and persistent skin inflammation.

Recent studies have identified cathepsin C (CTSC) as an important regulator of keratinocyte proliferation and inflammation. Dysregulation of CTSC promotes keratinocyte hyperproliferation through NF-κB–dependent mechanisms, linking protease activity to inflammatory transcriptional programs (85). This connection highlights how noncanonical regulators of epidermal biology can feed into NF-κB signaling to reinforce pathological growth patterns. Similarly, RSAD2, an interferon-stimulated gene traditionally associated with antiviral responses, has been implicated in psoriasis-associated keratinocyte hyperproliferation. Downregulation of RSAD2 ameliorates epidermal thickening and skin inflammation by suppressing the TAK1/NF-κB axis, underscoring the role of NF-κB as a shared downstream effector of inflammatory and stress-induced signals within keratinocytes (**Figure 9**) (86). These findings reinforce the concept that NF-κB signaling in keratinocytes is not limited to inflammatory cytokine production. Instead, NF-κB orchestrates a coordinated program that couples cell cycle progression, survival signaling, and inflammatory output, thereby creating self-sustaining loops of epidermal hyperplasia and inflammation.

### 5.2 Oxidative stress–inflammation coupling: SIRT1/Nrf2 as intrinsic brakes on NF-κB

Psoriatic lesions are characterized by elevated oxidative stress and metabolic perturbations, which further exacerbate inflammatory signaling. NF-κB sits at the intersection of oxidative stress and inflammation, responding dynamically to redox imbalance while simultaneously promoting the expression of proinflammatory mediators. Multiple lines of evidence indicate that SIRT1 functions as a critical negative regulator of NF-κB in keratinocytes (87, 88). Activation of SIRT1 suppresses NF-κB–dependent transcription by deacetylating key signaling components, thereby limiting inflammatory amplification. A comprehensive review of salidroside highlights its ability to activate SIRT1 and inhibit MAPK, NF-κB, and STAT3 signaling, collectively reducing oxidative stress and inflammatory burden in psoriasis (87). Complementary to SIRT1, the Nrf2 pathway serves as a master regulator of antioxidant defenses. Natural compounds such as tryptanthrin exert anti-psoriatic effects by concurrently modulating NF-κB, MAPK, and Nrf2 signaling pathways (89). This coordinated regulation illustrates how redox homeostasis and inflammatory control are tightly coupled within keratinocytes. Additional evidence supports the protective role of endogenous regulators in this context. LARP7 has been shown to alleviate psoriasis symptoms in experimental models by regulating the SIRT1/NF-κB axis, further emphasizing the importance of intrinsic inhibitory circuits that restrain excessive NF-κB activation (88).

Together, these findings support a model in which SIRT1 and Nrf2 function as metabolic and oxidative “braking systems” for NF-κB. Disruption of these brakes shifts keratinocytes toward a proinflammatory, hyperproliferative state, whereas their activation restores redox balance and dampens pathological NF-κB signaling.

### 5.3 AhR/NF-κB and environmental ligand–sensing immunomodulation

Keratinocytes are uniquely positioned at the interface between the host and the environment, constantly exposed to microbial products, xenobiotics, and endogenous metabolites. The aryl hydrocarbon receptor (AhR) serves as a key sensor of such environmental and metabolic ligands, enabling keratinocytes to translate external cues into transcriptional responses (90–92). Recent studies demonstrate that indigo, a natural AhR agonist, alleviates psoriasis-like inflammation through modulation of the AhR/NF-κB signaling axis (93). Activation of AhR attenuates NF-κB–dependent inflammatory gene expression, highlighting a crosstalk mechanism whereby ligand-activated transcription factors fine-tune NF-κB output. This interaction underscores a broader principle in psoriatic pathogenesis: keratinocyte-intrinsic NF-κB activity is dynamically shaped by environmental sensing pathways. By integrating signals from AhR, keratinocytes can modulate inflammatory tone in response to changes in the cutaneous microenvironment, providing an additional layer of regulation beyond immune-derived cytokines. Collectively, these keratinocyte-intrinsic programs demonstrate that NF-κB functions as a central regulator of epidermal pathology in psoriasis, coordinating hyperproliferation, oxidative stress responses, and inflammatory output. Rather than acting as passive recipients of immune signals, keratinocytes actively shape disease progression through NF-κB–dependent amplification loops (94–99). This intrinsic regulatory capacity positions keratinocytes as key drivers of psoriatic inflammation and highlights NF-κB as a critical hub linking immune activation to tissue-specific pathology.

## 6 Epigenetic and non-coding RNA regulation: tuning the NF-κB hub

While NF-κB integrates diverse upstream inflammatory signals, the magnitude, duration, and persistence of its transcriptional output are critically shaped by epigenetic and post-transcriptional regulatory layers. Non-coding RNAs and chromatin-associated modifiers act as key tuners of NF-κB signaling, enabling transient inflammatory cues to be converted into sustained pathogenic programs in psoriasis. This regulatory dimension is particularly relevant given the chronic and relapsing nature of the disease.

### 6.1 miRNAs controlling NF-κB activation and inflammatory tone

**Figure 10:**
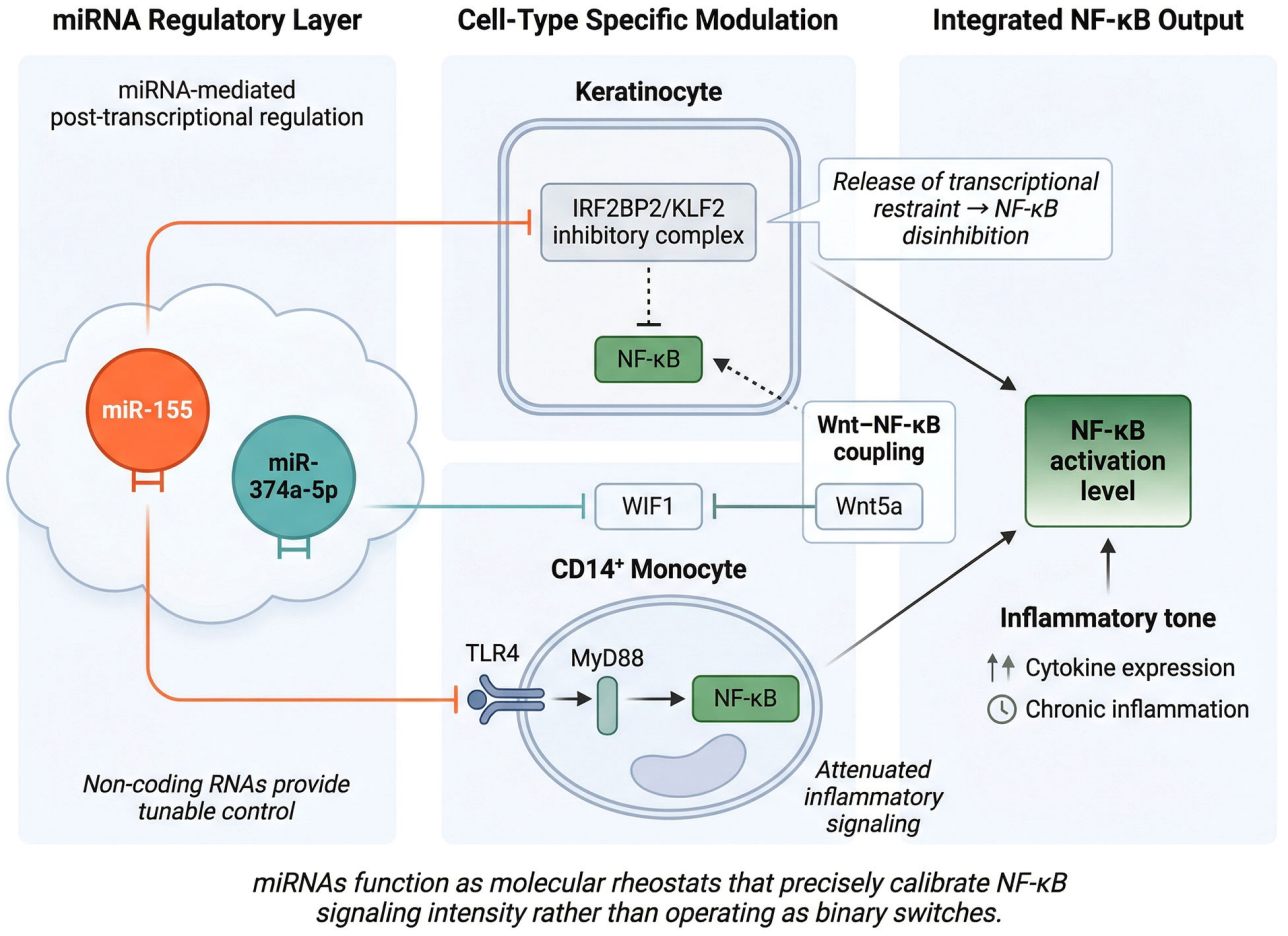
miRNA-mediated tuning of NF-κB inflammatory tone in psoriasis. MicroRNAs fine-tune NF-κB signaling by modulating inhibitory checkpoints and intersecting pathways in psoriasis. miR-155 amplifies NF-κB activity in keratinocytes by suppressing the IRF2BP2/KLF2 axis and reinforces inflammation in innate immune cells through TLR4/MyD88 signaling. In parallel, miR-374a-5p activates Wnt5a–NF-κB signaling via WIF1 downregulation. Together, these miRNAs act as rheostats that shape inflammatory intensity and disease chronicity.

MicroRNAs (miRNAs) represent a rapid and reversible layer of post-transcriptional regulation that fine-tunes inflammatory signaling pathways. In psoriasis, several miRNAs have been identified as critical modulators of NF-κB activity across both immune cells and keratinocytes. Among these, miR-155 is one of the most extensively studied proinflammatory miRNAs. In keratinocytes, miR-155 promotes inflammatory responses by targeting the IRF2BP2/KLF2 axis, thereby releasing NF-κB from transcriptional restraint and enhancing proinflammatory gene expression (100). This mechanism underscores how miR-155 amplifies NF-κB signaling indirectly by suppressing endogenous inhibitory pathways. The pathogenic role of miR-155 is not restricted to keratinocytes. In CD14^+^ monocytes, inhibition of miR-155 attenuates inflammatory responses and oxidative stress through suppression of the TLR4/MyD88/NF-κB pathway (101). These findings highlight miR-155 as a cross-compartmental regulator that reinforces NF-κB–dependent inflammation in both innate immune cells and epidermal compartments. Additional miRNAs contribute to NF-κB activation through distinct signaling routes. miR-374a-5p, for example, drives psoriasis pathogenesis by downregulating WIF1, leading to activation of Wnt5a/NF-κB signaling (102). This pathway illustrates how miRNAs can indirectly modulate NF-κB by intersecting with developmental and noncanonical inflammatory pathways (**Figure 10**).

Collectively, these studies demonstrate that miRNAs function as rheostats of NF-κB signaling, adjusting inflammatory tone rather than acting as binary switches. Dysregulation of these miRNA-mediated control mechanisms skews NF-κB activity toward persistent inflammation, contributing to disease chronicity.

### 6.2 lncRNAs integrating hypoxia, STAT3 signaling, and NF-κB

Long non-coding RNAs (lncRNAs) provide an additional layer of regulatory complexity by acting as molecular scaffolds, transcriptional modulators, or epigenetic guides. In psoriasis, emerging evidence indicates that lncRNAs serve as integrative nodes linking environmental stress, oncogenic-like signaling pathways, and inflammatory transcriptional programs. The lncRNA UCA1 exemplifies this integrative role. UCA1 promotes keratinocyte-driven inflammation by suppressing METTL14, thereby altering RNA methylation dynamics and enhancing HIF-1α/NF-κB signaling (103). This mechanism couples hypoxia-associated stress responses with inflammatory amplification, providing a molecular explanation for how metabolic and microenvironmental perturbations reinforce NF-κB activity in psoriatic lesions. Similarly, lncRNA-GDA-1 has been shown to promote keratinocyte proliferation and inflammation by regulating the FOXM1/STAT3/NF-κB axis (104). Through this pathway, lncRNA-GDA-1 integrates proliferative and inflammatory signaling, reinforcing epidermal hyperplasia and immune activation. These examples highlight a unifying principle: lncRNAs act as signal integrators that bridge upstream stress and growth signals with NF-κB–dependent transcription (105–107). By coordinating multiple signaling axes, lncRNAs enable keratinocytes to sustain inflammatory programs even in the absence of continuous external stimulation.

### 6.3 Chromatin and DNA/RNA modification layers shaping NF-κB persistence

Beyond non-coding RNAs, chromatin modifiers and nucleic acid–modifying enzymes exert long-lasting effects on NF-κB signaling by altering transcriptional accessibility and epigenetic memory. Given that NF-κB functions as a transcriptional hub, such epigenetic regulation is particularly important for determining whether inflammatory responses are transient or persist over time.

Evidence from pharmacological and genetic studies indicates that EZH2, a core component of the Polycomb repressive complex 2, contributes to psoriatic inflammation through modulation of NF-κB–associated transcriptional programs. Treatment with QingReDu capsule ameliorates psoriasis vulgaris by regulating the EZH2/NF-κB signaling pathway, suggesting that epigenetic repression and inflammatory activation are tightly interconnected (108). In parallel, DNA demethylation pathways also influence NF-κB activity. Transglutaminase 3 (TGM3) has been shown to attenuate skin inflammation by inhibiting NF-κB activation through phosphorylated STAT3–TET3 signaling (109). This pathway links transcription factor activation to DNA demethylation, providing a mechanism by which inflammatory signals can be epigenetically encoded or erased. Together, these findings support the concept that epigenetic modifiers act as molecular memory keepers of NF-κB signaling. By shaping chromatin accessibility and nucleic acid modification states, these regulators determine the persistence of NF-κB–driven inflammatory programs in psoriasis.

Non-coding RNAs and epigenetic regulators constitute a critical regulatory layer that tunes NF-κB signaling in psoriasis. By modulating transcriptional thresholds, signal duration, and inflammatory memory, these mechanisms enable transient immune and stress signals to be converted into sustained pathological inflammation. Recognizing NF-κB as a transcriptional hub highlights why epigenetic and post-transcriptional regulation are essential determinants of disease chronicity and therapeutic responsiveness. **Table 3** captures emerging epigenetic and post-transcriptional regulatory layers that fine-tune NF-κB activity and contribute to inflammatory persistence.

**Table 3:** Epigenetic and post-transcriptional tuning of NF-κB signaling in psoriasis.

Regulatory layer	Molecule	Target/pathway	Effect on NF-κB	Functional outcome	Representative PMID(s)
miRNA	miR-155	IRF2BP2/KLF2	Disinhibition	KC & immune inflammation	PMID: 39219281
miRNA	miR-155 inhibition	TLR4/MyD88	Suppression	Reduced monocyte inflammation	PMID: 35173453
miRNA	miR-374a-5p	WIF1 ↓ → Wnt5a	Activation	Chronic inflammatory tone	PMID: 38604345
lncRNA	UCA1	METTL14 ↓ → HIF-1α	Activation	Hypoxia-linked inflammation	PMID: 37076497
lncRNA	GDA-1	FOXM1 → STAT3	Activation	KC proliferation	PMID: 36943641
Epigenetic	EZH2	Chromatin remodeling	NF-κB facilitation	Sustained transcription	PMID: 40029458; PMID: 33011750
DNA modification	TGM3–STAT3–TET3	DNA demethylation	NF-κB suppression	Anti-inflammatory effect	PMID: 35545140

## 7 Therapeutic targeting of the NF-κB convergence hub: opportunities and challenges

Given its central role in integrating innate and adaptive immune signals, NF-κB represents an attractive yet challenging therapeutic target in psoriasis. Rather than acting as a single linear pathway, NF-κB functions as a convergence hub shaped by upstream sensors, adaptor complexes, and tissue-specific modulators. Recent therapeutic strategies increasingly aim to exploit this network architecture by targeting discrete nodes within the NF-κB signaling framework, thereby attenuating pathogenic inflammation while minimizing systemic immune suppression.

### 7.1 Direct pathway antagonism: upstream sensors and TRAF/IKK modulation

One emerging strategy focuses on upstream innate immune sensors that feed into NF-κB activation. Targeting such entry points offers the advantage of intercepting inflammatory signaling before it is broadly amplified across immune and epidermal compartments. The STING antagonist H-151 exemplifies this approach. By suppressing STING-dependent NF-κB activation, H-151 effectively ameliorates psoriasis-like inflammation in experimental models. Importantly, this intervention selectively dampens nucleic acid–driven inflammatory signaling without globally inhibiting NF-κB activity, highlighting the potential of upstream sensor modulation to achieve therapeutic specificity. In parallel, targeting adaptor-level signaling nodes has gained increasing attention. The TRIM14/TRAF3/NF-κB axis represents a critical regulatory module linking innate immune signaling to inflammatory transcriptional programs. A novel delivery strategy employing cannabidiol-loaded hydrogel microneedle patches has been shown to inhibit this axis, resulting in reduced skin inflammation (110). This approach underscores two key advantages: first, selective interference with adaptor-mediated NF-κB activation; and second, localized cutaneous delivery, which confines immunomodulation to lesional skin and reduces the risk of systemic toxicity.

Beyond broad suppression of downstream inflammatory signaling, several upstream- and subunit-selective strategies may provide more precise approaches for modulating NF-κB-centered inflammation in psoriasis. First, inhibition of TLR7 or modulation of TLR7 trafficking may attenuate innate immune entry into the NF-κB network before downstream amplification occurs (25, 30). In this regard, SHP2-dependent regulation of TLR7 trafficking provides a mechanistically attractive target, because pharmacological SHP2 inhibition has been shown to reduce aberrant TLR7-driven inflammatory signaling in psoriasis-like inflammation (30). Second, targeting specific NF-κB subunits, such as c-Rel, may allow selective disruption of pathogenic immune-cell transcriptional programs while preserving broader RelA/p65-dependent homeostatic functions (43). Third, adaptor-level modulation at TRAF or TAK1/IKK nodes may provide an intermediate strategy between receptor-level blockade and global NF-κB inhibition (31, 33). Together, these approaches support a shift from non-specific NF-κB inhibition toward pathway-, subunit-, and cell-type-selective immunomodulation in psoriasis.

### 7.2 Repurposed and pleiotropic small molecules targeting NF-κB networks

Another promising avenue involves the repurposing of clinically approved or well-characterized small molecules with pleiotropic anti-inflammatory properties. Such agents often target NF-κB indirectly by modulating parallel signaling pathways that converge on inflammatory transcriptional programs. Nintedanib, a multi-tyrosine kinase inhibitor originally developed for fibrotic diseases and cancer, has demonstrated efficacy in ameliorating imiquimod-induced psoriasis in mice by inhibiting both NF-κB and VEGFR2 signaling (111). This dual inhibition highlights the importance of vascular–inflammatory coupling in psoriasis, as angiogenesis and inflammation mutually reinforce lesion persistence and expansion. By simultaneously targeting inflammatory signaling and pathological vascular remodeling, pleiotropic agents such as nintedanib exemplify how NF-κB–centered networks can be therapeutically modulated without direct NF-κB blockade. This indirect strategy may offer improved safety profiles compared with global NF-κB inhibition. To facilitate comparison of these therapeutic approaches, **Table 4** summarizes the major upstream sensors, adaptor-level targets, and pleiotropic small-molecule strategies discussed in Sections 7.1 and 7.2, with emphasis on their NF-κB-related mechanisms, experimental contexts, and translational implications.

**Table 4:** Summary of therapeutic strategies targeting NF-κB-associated pathways.

Therapeutic strategy/agent	Main target or pathway	NF-κB-related mechanism	Experimental context	Translational implication	Representative PMID(s)
STING antagonist H-151	STING–NF-κB axis	Suppresses STING-dependent NF-κB activation and reduces inflammatory cytokine production	IMQ-induced psoriasis-like inflammation	Supports selective inhibition of upstream nucleic acid-sensing pathways rather than global NF-κB blockade	PMID: 34460100
cGAS–STING pathway inhibitors/nanoinhibitors	cGAS–STING signaling	Reduces cytosolic DNA-induced inflammatory signaling and downstream NF-κB-associated responses	Psoriasis-like experimental models	Suggests that targeting DNA-sensing pathways may attenuate innate inflammatory amplification	PMID: 38017308; PMID: 40174660
TLR7 pathway antagonism	TLR7-dependent innate immune activation	Limits upstream TLR7-mediated NF-κB activation	TLR7/IMQ-related inflammatory models	Provides a rationale for targeting TLR7 as an upstream inflammatory entry point	PMID: 41708477
SHP2-mediated regulation of TLR7 trafficking	TLR7 trafficking/receptor localization	Modulates TLR7 localization and downstream inflammatory signaling	IMQ-induced psoriasis-like inflammation	Highlights receptor trafficking as a targetable regulatory layer upstream of NF-κB	PMID: 34936223
Cannabidiol-loaded hydrogel microneedle patch	TRIM14/TRAF3/NF-κB axis	Inhibits adaptor-level NF-κB activation through localized skin delivery	Psoriasis-like inflammation model	Combines pathway modulation with skin-localized delivery to reduce systemic exposure	PMID: 39933682
Dimethyl fumarate	IKKα/β, MSK1, RSK1/2, NF-κB/p65	Reduces NF-κB/p65 activation and inflammatory signaling	Psoriasis-related immune cells/clinical treatment context	Illustrates pharmacological modulation of NF-κB-associated kinase networks	PMID: 32309199
Amlexanox	Th17 cells and NF-κB signaling	Suppresses Th17-associated inflammation and NF-κB signaling	IMQ-induced psoriasis-like dermatitis	Supports repurposed anti-inflammatory modulation of NF-κB-associated networks	PMID: 39983433
Nintedanib	VEGFR2 and NF-κB signaling	Inhibits vascular-inflammatory coupling and NF-κB-associated inflammation	IMQ-induced psoriasis-like mouse model	Suggests that pleiotropic agents may target both inflammation and angiogenesis in psoriasis	PMID: 34547680

### 7.3 Natural products and traditional medicine: multi-target suppression of NF-κB networks

Natural compounds and traditional medicinal formulations provide a rich source of multi-target modulators capable of fine-tuning NF-κB–centered inflammatory networks. Rather than inhibiting a single molecular node, these agents often act across interconnected signaling pathways, thereby restoring immune balance. Several examples illustrate this principle. Indigo alleviates psoriasis-like inflammation through modulation of the AhR/NF-κB axis, integrating environmental ligand sensing with inflammatory control (93). Tryptanthrin exerts anti-psoriatic effects by simultaneously regulating NF-κB, MAPK, and Nrf2 pathways, thereby coupling anti-inflammatory and antioxidant responses (89). Benzoylpaeoniflorin has been shown to suppress psoriasis-like inflammation by restoring immune balance and inhibiting NF-κB signaling (112). Traditional formulations such as QingReDu capsule further expand this concept by targeting both epigenetic regulators and inflammatory pathways, including the EZH2/NF-κB axis (108). Such multi-layered regulation is particularly relevant for chronic inflammatory diseases like psoriasis, where sustained NF-κB activity is reinforced by epigenetic and transcriptional memory.

Together, these studies support the notion that network-level modulation, rather than single-target inhibition, may be especially effective in attenuating NF-κB–driven pathology.

### 7.4 Key translational considerations and future challenges

Despite compelling preclinical evidence, several challenges must be addressed to translate NF-κB–targeted strategies into clinical practice. First, systemic inhibition of NF-κB carries inherent safety concerns, given its essential roles in host defense and tissue homeostasis. This limitation highlights the importance of localized delivery approaches, such as topical formulations, microneedle systems, or skin-targeted nanoparticles, to confine therapeutic effects to diseased tissue (113–118). Second, cell type–specific targeting remains a critical consideration. NF-κB signaling fulfills distinct functions in keratinocytes, dendritic cells, and Th17 cells. Therapeutic strategies that preferentially modulate keratinocyte-intrinsic NF-κB amplification may differ fundamentally from those aimed at suppressing immune cell–driven inflammation. Understanding these distinctions will be essential for optimizing treatment efficacy while minimizing adverse effects. Finally, biomarker-driven patient stratification may enhance the precision of NF-κB–centered therapies. Differential expression or activation of upstream sensors and downstream effectors—such as elevated STING activity, c-Rel–dominant NF-κB programs, or enhanced IL-36 signaling—could inform personalized therapeutic selection. Integrating molecular biomarkers with clinical phenotyping may enable rational deployment of NF-κB–targeted interventions in specific patient subsets.

Taken together, these therapeutic studies illustrate both the promise and complexity of targeting NF-κB as a convergence hub in psoriasis. Rather than a single druggable pathway, NF-κB represents a networked signaling architecture that can be modulated at multiple levels. Future therapeutic success will likely depend on combining selective pathway interception, localized delivery, and biomarker-guided stratification to achieve durable disease control with acceptable safety profiles.

## 8 Conclusions and future perspectives

Psoriasis arises from the dynamic interplay between innate immune activation, adaptive immune maintenance, and keratinocyte-intrinsic amplification. In this review, we highlight NF-κB as a convergence hub that integrates these diverse inflammatory inputs into sustained transcriptional programs. Rather than acting as a linear pathway, NF-κB functions as a networked signaling architecture shaped by upstream sensors, adaptor modules, transcriptional subunits, and epigenetic regulators. This systems-level perspective provides a unifying framework for understanding how transient immune and stress signals are converted into chronic, tissue-confined inflammation.

### 8.1 Cell-type–resolved mapping of NF-κB programs

A critical future direction lies in resolving cell type–specific NF-κB activity within psoriatic skin. Keratinocytes, dendritic cells, and Th17 cells each employ NF-κB signaling to fulfill distinct functional roles, ranging from inflammatory amplification to immune orchestration. Advances in single-cell and spatial transcriptomics, coupled with chromatin accessibility and transcription factor occupancy analyses, offer unprecedented opportunities to map NF-κB–dependent gene programs at cellular and spatial resolution. Such approaches will enable the identification of compartment-specific NF-κB signatures and clarify how intercellular communication sustains inflammation within localized lesions.

### 8.2 Subunit-specific targeting of NF-κB

Another promising avenue is the development of subunit-specific NF-κB targeting strategies. Emerging evidence suggests that distinct NF-κB subunits, particularly c-Rel, play nonredundant roles in psoriasis by selectively driving pathogenic inflammatory programs. Compared with global NF-κB inhibition, targeting specific subunits may provide improved therapeutic precision and safety by sparing essential homeostatic functions. Further mechanistic dissection of subunit-dependent transcriptional networks will be crucial for translating this concept into clinically viable interventions.

### 8.3 Combination strategies to fine-tune NF-κB networks

Given the central position of NF-κB within inflammatory networks, combination therapeutic strategies are likely to offer superior efficacy compared with single-agent approaches. In particular, integrating established IL-17–targeted therapies with modulators of NF-κB regulatory circuits—such as SIRT1, Nrf2, or AhR pathways—may allow inflammatory responses to be attenuated while preserving immune balance. Such combinatorial approaches could reduce treatment resistance, improve durability of response, and address disease heterogeneity among patients.

### 8.4 Next-generation delivery systems for localized immunomodulation

Finally, the success of NF-κB–centered therapies will depend heavily on innovative drug delivery technologies. Next-generation platforms, including microneedle patches, hydrogel-based systems, and nanoparticle formulations, offer the potential to deliver immunomodulatory agents directly to the skin. By restricting drug exposure to lesional tissue, these approaches may mitigate systemic toxicity and enable more aggressive modulation of NF-κB–associated pathways. The integration of advanced delivery systems with molecular targeting strategies represents a critical step toward precision dermatological therapy.

### 8.5 Perspective

Together, these future directions underscore the importance of viewing NF-κB not merely as a proinflammatory pathway, but as a context-dependent regulatory hub that orchestrates immune–epidermal interactions in psoriasis. By combining cell type–resolved analysis, subunit-specific targeting, rational combination therapies, and advanced delivery technologies, it may be possible to transform NF-κB from a historically “undruggable” target into a precision-modulated therapeutic nexus. Such advances hold promise for achieving durable disease control while minimizing adverse effects, ultimately improving outcomes for patients with psoriasis.
